# Eruption pattern of the maxillary canines: features indicating treatment needs as seen in PTG at the late mixed stage—Part II

**DOI:** 10.1007/s40368-022-00719-5

**Published:** 2022-06-10

**Authors:** J. Ristaniemi, T. Karjalainen, K. Kujasalo, W. Rajala, P. Pesonen, R. Lähdesmäki

**Affiliations:** 1 Research Unit of Oral Health Sciences, Oral Development and Orthodontics, Faculty of Medicine, University of Oulu, Oulu, Finland; 2 Infrastructure for Population Studies, Faculty of Medicine, University of Oulu, Oulu, Finland; 3 Oral and Maxillofacial Department, Medical Research Center Oulu (MRC Oulu), Oulu University Hospital, Oulu, Finland

**Keywords:** Dental age, Developing dentition, Root formation, Interceptive treatment, Headgear, Space conditions

## Abstract

**Aim:**

To describe features of maxillary permanent canines in the late mixed stage as seen in panoramic radiograph (PTG) that later needed treatment to erupt into the oral cavity and to compare them with naturally erupted canines.

**Methods:**

The cross-sectional part of this retrospective register-based study consisted of 1454 PTGs of children (mean age 9.3 years) living in Eastern Finland, while the longitudinal part involved patient data on 184 treated maxillary canines. The variables examined were treatment needs, overlapping and inclination of the maxillary canines, the development stage of the canines and lateral incisors and dental age.

**Results:**

Only 11.6% of the maxillary canines needed treatment, including interceptive procedures. The treated canines significantly more often had overlapping (*p* < 0.001), larger inclination (*p* = 0.001) and incomplete lateral incisors (*p* = 0.002) than did the naturally erupted canines. The children treated significantly more often had a delayed dental age (p = 0.035). Clear overlapping was closely associated with all treatment modalities, whereas some overlapping and a large inclination angle (≥ 25°) were associated especially in cases of late treatment. An incomplete lateral incisor and delayed dental age were associated with treatment.

**Conclusion:**

An association with treatment needs was found especially in the case of overlapping and a large inclination angle of the maxillary canine a couple of years before eruption into the oral cavity. These features can be early signs and indications for instant or later treatment of a maxillary canine and underline the importance of monitoring space conditions and erupting canines.

**Supplementary Information:**

The online version contains supplementary material available at 10.1007/s40368-022-00719-5.

## Introduction

Maxillary canines have a multi-stage eruption route, and disturbances in their eruption are common clinical problems affecting the developing permanent dentition, with a prevalence of about 1.7% (Ericson and Kurol 1986). Disturbances in the eruption of maxillary canines can cause major complications such as root resorption of an adjacent tooth (Ericson and Kurol 2000; Hadler-Olsen et al. 2015). Furthermore, late treatment for displaced canines is usually a long-term, expensive procedure (Bazargani et al. 2013). Thus, early diagnosis is crucial. Alongside clinical inspection and palpation, the primary routine radiographic examination for assessing the developing dentition and its erupting maxillary canines is panoramic radiograph (PTG), which enables diagnosis of maxillary canine displacement from the age of eight years onwards (Sajnani and King 2012a) by means of geometric measurements, as first defined by Ericson and Kurol (1988).

In the earlier stages of eruption and development, a maxillary canine is often seen in PTG to overlap with the lateral incisor (Ristaniemi et al. 2022). The age of 8–9 years seems to be the turning point after which overlapping will decrease if the canine erupts normally (Fernández et al. 1998; Sajnani and King 2012a), whereas in abnormal eruption overlapping may increase (Sajnani and King 2012a). Sectors reflecting the magnitude of such overlapping have been said to be better predictors of displacement than the inclination of the maxillary canine itself (Warford et al. 2003).

The inclination of a normally erupting maxillary canine has been shown in earlier studies to increase mesially until the age of 8–9 years after which the tooth progressively uprights itself (Fernández et al. 1998; Sajnani and King 2012a), whereas in an ectopic eruption path it will still be pronouncedly inclined after age of nine years (Sajnani and King 2012a; Chalakkal et al. 2011). Inclination during natural eruption has shown to decrease significantly as root development in the maxillary canine continues from one-third to halfway (Ristaniemi et al. 2022).

Root development in a permanent maxillary canine will mainly (84.2%) have reached one-third to half of the final length by the age of 8.5–10.5 years (Ristaniemi et al. 2022) and will usually be almost complete by the time the tooth erupts into the oral cavity (Nolla 1960; Haavikko 1970). Root development in the maxillary canines during natural eruption has been shown to be more advanced in girls (Ristaniemi et al. 2022), while no difference has been found between impacted and normally erupting canines (Sajnani and King 2012a).

It is notable that the maxillary lateral incisors are still mainly (78.5%) incomplete in children aged 8.5–10.5 years, with girls having complete lateral incisors more often than boys at this age (Ristaniemi et al. 2022). It has been shown earlier that overlapping of a maxillary canine with a lateral incisor can be an early sign of abnormal eruption of the canine if the development of the lateral incisor is already complete (Fernández et al. 1998).

Dental age as assessed from a PTG by Demirjian’s method (Demirjian et al. 1973; Demirjian and Goldstein 1976) has been reported earlier to be more advanced in girls at the late mixed dentition stage (Demirjian and Levesque 1980; Chaillet et al. 2004). In addition, late timing of dental development has been related to larger inclination angles of the maxillary canines during their eruption (Ristaniemi et al. 2022) and to displacement of the maxillary canines (Rozylo-Kalinowska et al. 2011; Naser et al. 2011). Nevertheless, the results of Sajnani and King (2012b) showed that less than half of the impacted canines could be attributed to delayed dental development.

If eruption disturbance in a maxillary canine can be diagnosed early, interceptive treatment is usually sufficient, and no actual impaction will occur. According to many earlier studies, extraction of primary maxillary canine at an age of 10–13 years is an effective treatment for a palatally displaced permanent canine (Ericson and Kurol 1988; Power and Short 1993; Baccetti et al. 2008; Bazargani et al. 2014; Naoumova et al. 2015). Use of headgear has been shown to have a positive effect on the eruption path of maxillary canine (Baccetti et al. 2008; Armi et al. 2011), especially at an early stage in the mixed dentition (Silvola et al. 2009; Hadler-Olsen et al. 2018).

The aim of this work was to describe the features of maxillary permanent canines seen in PTG a couple of years before their expected eruption into the oral cavity (Eskeli et al. 1999) that may later result in a need for treatment in order to ensure eruption. Our hypothesis was that the degree of overlapping of a canine crown with a lateral incisor root and mesial inclination of a canine will occur more prominently a couple of years before eruption in maxillary canines that will need treatment later. A further hypothesis was that the maxillary canine and lateral incisor are at earlier stages of root development and dental age is late in children with treated maxillary canine(s) a couple of years before the time of eruption than in those with naturally erupted maxillary canines (no treatment).

## Materials and methods

The material for this cross-sectional and longitudinal retrospective register-based study consisted of 1454 PTGs of the developing permanent dentition, mainly in third-year primary school children born between 1980 and 1996. This material was gathered at a health centre in Eastern Finland, the PTGs having been taken by the centre’s radiological department during annual oral check-ups to examine the development of the permanent dentition. The PTGs were copied digitally and basic background information was gathered during the years 2006–2007 by TK and RL. Dental records and other dental radiographs were examined by RL, JR, KK and WR during the years 2016–2020. More specific descriptions of the subjects and the variables studied are given in part I of this study (Ristaniemi et al. 2022).

### Overlapping and inclination

The classifications of the maxillary canine crowns with respect to overlapping with the lateral incisor root (Grades 0–2) (overlapping) and inclination (α) (inclination) were assessed from the PTGs by TK, using the neaView Radiology software (Neagen Oy) (see Ristaniemi et al. 2022, modified from Ericson and Kurol 1988). The grades of overlapping were Grade 0 (no overlapping), Grade 1 (crown of the canine covering half or less of the width of the lateral incisor root) and Grade 2 (crown of the canine covering more than half of the width of the lateral incisor root). The inclination was measured as the angle between the canine axis and the midsagittal suture of the maxilla.

### Developmental stages of maxillary canines and lateral incisors

The development stage of each maxillary canine root was assessed from the PTGs by WR using the method of Nolla (1960) on a scale of: Stage 1 (root formation started, Nolla’s value < 7.0), Stage 2 (one-third of the root length completed, Nolla’s value 7.0 or 7.2), Stage 3 (half of the root length completed, Nolla’s value 7.5 or 7.7), Stage 4 (at least two-thirds of the root length completed, Nolla’s value 8.0–8.7) and Stage 5 (root completed, apex open/closed, Nolla’s value 9.0–10.0) (see Ristaniemi et al. 2022). The development stages of the maxillary lateral incisors were assessed by WR from the PTGs simultaneously with those of the canines and grouped by reference to Nolla (1960) into incomplete (Nolla’s value 8.7 or less) or complete (Stage 5, Nolla’s value 9.0–10.0).

### Dental age

Dental age was analysed from the PTGs by EM and JI using Demirjian’s method, in which seven teeth in the third quadrant are scored (Demirjian et al. 1973; Demirjian and Goldstein 1976) and then assessed by reference to Finnish maturity curves (Chaillet et al. 2004). The child’s dental age was considered normal with a mean ± 1SD (1SD = 1 year). For the present purposes, the dental ages were grouped into normal (early/normal) and delayed relative to the subject’s chronological age (Eskeli et al. 1999).

### Need for treatment

**Fig. 1:**
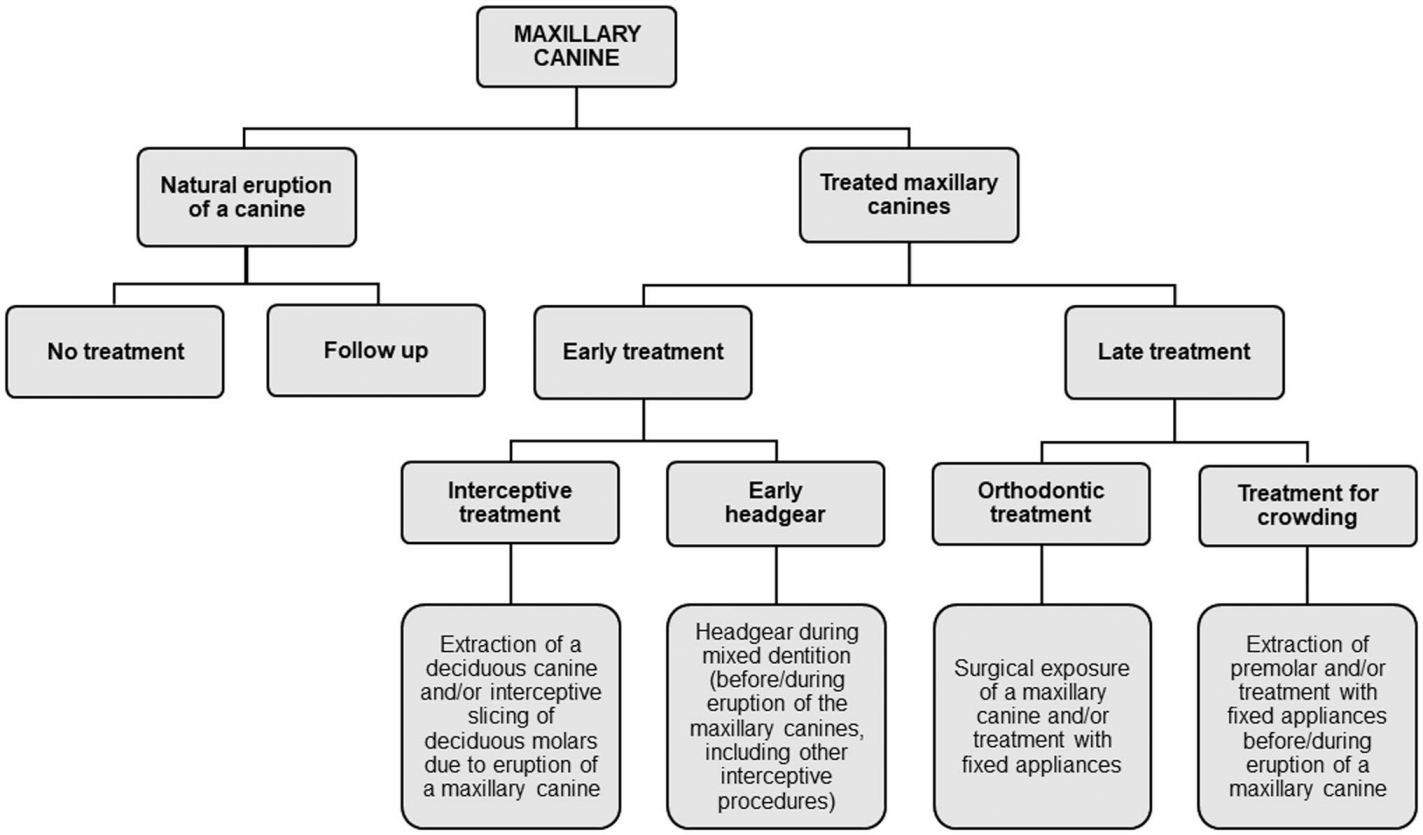
Treated and naturally erupted maxillary canines categorised according to treatment need

The need for treatment because of eruption of a maxillary canine was determined from the dental records by RL, JR and KK. These included patient records for the time after PTG and until the maxillary canine had erupted in all cases for which such records could be found in the health centre’s paper archives or software data. Orthodontic treatment before PTG or after the eruption of the maxillary canine concerned was not included. The need for treatment was defined and categorised (Fig. 1).

This report is focused on the maxillary canines that needed early or late treatment in order to erupt into the oral cavity, referred to below as “treated canines”. The inclusion criteria for this group were chronological age 8.5–10.5 at the time of the PTG, no missing teeth, no peg-shaped lateral incisors, no syndromes or clefts but maxillary canine(s) that needed treatment. The group with “natural eruption of a canine” as defined in part I (Ristaniemi et al. 2022) was used for comparison.

### Statistics

The statistical analyses were carried out using SPSS (version 26.0, IBM SPSS Statistics, Armonk, NY, USA) and SAS Enterprise guide 7.1. *P* values < 0.05 were considered statistically significant. The reliability of the assessments was estimated using Cohen’s kappa or intra-class correlation (ICC) (see “Statistics” in Ristaniemi et al. 2022). The normality of the continuous variables was assessed visually using histograms, and thus gender-specific mean ages (chronological and dental) were analysed with the independent samples *t* test.

Comparisons between the genders and the variables examined were conducted with the Pearson Chi-square test or Fisher’s exact test, as were comparisons between overlapping and inclination, stages of development in the maxillary canine, stages of development in the lateral incisor and dental age. Comparisons between the treated and naturally erupted maxillary canines were evaluated using Pearson Chi-square test. Comparisons between inclination and stages of development in the maxillary canine and lateral incisor, and also dental age were conducted with Fisher’s Exact test, as were the examinations of the differences between the need treatment groups and the categories of overlapping and inclination, the stage of root development in the maxillary canine and the lateral incisor, and the dental age.

The single associations of independent variables with the response variables were determined with crude logistic regression models for the genders separately, while adjusted logistic regression models were used to check the associations of all independent variables to the response variables. Statistically significant two-way interaction terms were included in the final models. The strength of each association was illustrated with an odds ratio and 95% confidence interval. The logistic regression models were resolved using the SAS glimmix procedure with random effect to take account of children having two canines in the data.

### Ethics

The data were gathered retrospectively from the clinical dental records. According to Finnish legislation, permission for collecting information from a register was given by the keeper of that register (see “Ethics” in Ristaniemi et al. 2022). All personal information was coded for the analyses by RL to prevent identification.

## Results

### Reliability

Reliabilities were calculated for the assessments of overlapping, inclination, root development stage of the canine and dental age (see “Reliability” in Ristaniemi et al. 2022). The assessments provided to be reliable in terms of their repeatability.

### Descriptive statistics

The total material consisted of 1454 PTGs, including 2907 maxillary canines (one congenitally missing right maxillary canine), the mean age of the children being 9.3 years (SD 0.6) at the time of PTG. The need for treatment could be assessed in the case of 1962 maxillary canines, whereas only 11.6% (*n* = 228) of the canines actually needed treatment.

**Fig. 2:**
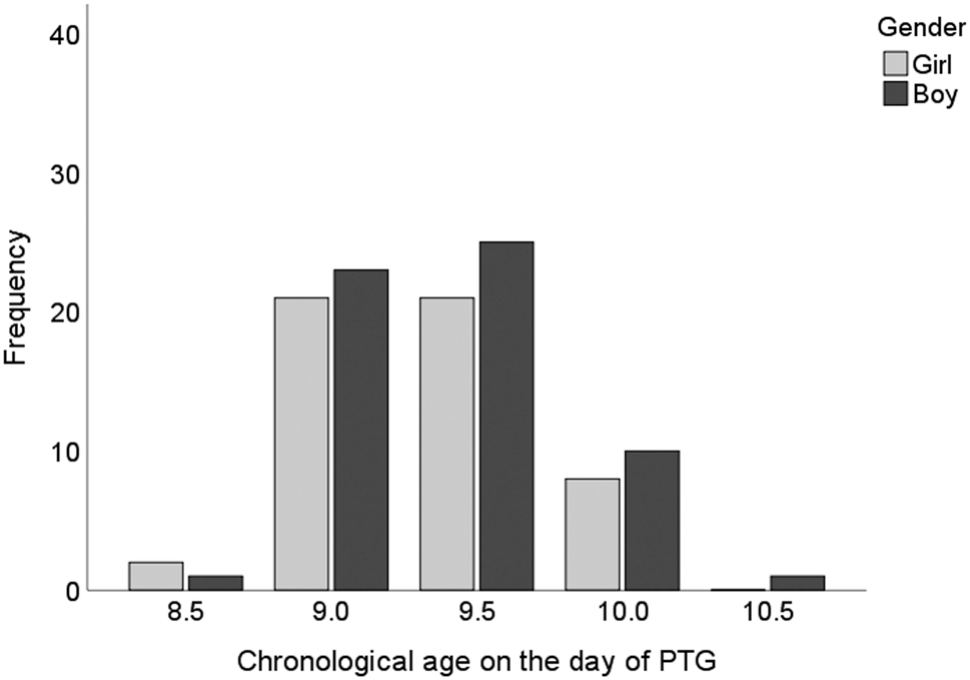
Distribution of children with treated maxillary canine(s) by chronological age

The inclusion criteria for treated canines were met by 112 PTGs (52 girls and 60 boys), including 184 maxillary canines, the mean age of this group (*n* = 112 children) at the time of PTG being 9.4 years (SD 0.4) (Fig. 2), with no difference between the genders (*p* = 0.996). Most common treatment need was for crowding (40.2%, n = 74), which more often affected boys (boys 59.5%, girls 40.5%). A third (*n* = 58) of the canines were treated by means of early headgear and for a fifth (*n* = 38) interceptive treatment was enough. Only 7.6% (*n* = 14) of the canines needed orthodontic treatment. There was no statistical difference between the genders with regard to the need for treatment (*n* = 0.481).

### Overlapping and inclination

Overlapping could be assessed for 178 treated canines. Some overlapping (Grade 1) was detected in half (*n* = 94) of the canines and clear overlapping (Grade 2) in 12.4% (*n* = 22), with no gender difference (*p* = 0.740). Inclination was detected in 179 treated canines, the mean angle being 14.4° (SD 9.0), while 11.7% (*n* = 21) had a large inclination angle (≥ 25). The grouped inclination angles did not differ statistically significantly between the genders (*p* = 0.062). The treated canines had overlapping significantly more often than the naturally erupting canines (*p* < 0.001) and also had a larger inclination (*p* = 0.001) (Table 1).

**Table 1:** Distribution of variables in the total material of PTGs, between naturally erupted and treated maxillary canines

	Treated canines	Naturally erupted canines^e^	Total material^e^
	*n *(%)	*n *(%)	*n *(%)
Overlapping of canine^a^			
Grade 0	62 (34.8)	798 (56.2)	1239 (54.5)
Grade 1	94 (52.8)	594 (41.8)	964 (42.4)
Grade 2	22 (12.4)	28 (2.0)	72 (3.2)
Total	178 (100.0)	1420 (100.0)	2275 (100.0)
*P* value^d^	< 0.001		
Inclination of canine (°)			
< 15	107 (59.8)	938 (65.9)	1493 (65.0)
15–19.9	26 (14.5)	273 (19.2)	430 (18.7)
20–24.9	25 (14.0)	134 (9.4)	234 (10.2)
≥ 25	21 (11.7)	78 (5.5)	139 (6.1)
Total	179 (100.0)	1423 (100.0)	2296 (100.0)
*P* value^d^	0.001		
Canine root development^b^			
Stage 1	16 (8.7)	93 (6.3)	314 (10.8)
Stage 2	115 (62.5)	856 (57.6)	1558 (53.8)
Stage 3	41 (22.3)	396 (26.6)	630 (21.8)
Stage 4	12 (6.5)	142 (9.5)	393 (13.6)
Total	184 (100.0)	1487 (100.0)	2895 (100.0)
*P* value^d^	0.172		
Lateral incisor development^b^			
Incomplete	160 (88.4)	1146 (78.5)	2196 (77.8)
Complete	21 (11.6)	314 (21.5)	626 (22.2)
Total	181 (100.0)	1460 (100.0)	2822 (100.0)
*P* value^d^	0.002		
Dental age^c^			
Normal	91 (90.1)	673 (95.2)	1202 (92.5)
Delayed	10 (9.9)	34 (4.8)	97 (7.5)
Total	101 (100.0)	707 (100.0)	1299 (100.0)
*P* value^d^	0.035		

^a^Grade 0 (no overlapping), Grade 1 (≤ ½ overlapping) and Grade 2 (> ½ overlapping)

^b^Division is based on developmental stages as defined by Nolla’s method (1960)

^c^Dental age is assessed by Demirjian’s method (Demirjian et al. 1973; Demirjian and Goldstein 1976)

^d^Pearson’s Chi-square test, significances are of differences between treated and naturally erupted maxillary canines

^e^Ristaniemi et al. (2022)

Crude logistic regression analysis showed that clear overlapping (OR = 8.10, 95% CI 3.94–16.47) and some overlapping (OR = 1.91, 95% CI 1.32–2.78) were associated with treated canines regardless of gender (Table 2). Only clear overlapping was associated with early treatment (Online Appendix 1); whereas in the late treatment group, both clear overlapping and some overlapping were associated with treatment (Online Appendix 2).

**Table 2:** Crude logistic regression analysis of associations between the independent variables and all treatments by gender in the treated maxillary canines

	Girls		Boys		All	
	OR	95% CI	OR	95% CI	OR	95% CI
Overlapping of canine^a^ (ref. Grade 0)						
Grade 1	**2.02**	**1.18–3.46**	**1.80**	**1.07–3.03**	**1.91**	**1.32–2.78**
Grade 2	**5.72**	**2.13–15.36**	**12.23**	**4.20–35.62**	**8.10**	**3.94–16.47**
Inclination of canine (°) (ref. < 15)						
15–19.9	0.93	0.46–1.88	0.96	0.50–1.83	0.95	0.59–1.52
20–24.9	1.78	0.83–3.82	1.00	0.43–2.32	1.37	0.78–2.40
≥ 25	**3.73**	**1.67–8.34**	1.25	0.45–3.48	**2.41**	**1.31–4.44**
Canine root development^b^ (ref. Stage 4)						
Stage 1	*^d^	*	1.79	0.47–6.79	1.49	0.58–3.82
Stage 2	*	*	1.34	0.41–4.41	1.52	0.75–3.09
Stage 3	*	*	0.72	0.20–2.68	1.06	0.50–2.27
Lateral incisor development^b^ (ref. complete)						
Incomplete	**2.51**	**1.18–5.37**	1.67	0.74–3.77	**2.05**	**1.18–3.56**
Dental age^c^ (ref. normal)						
Delayed	2.58	0.96–6.90	1.86	0.59–5.81	**2.29**	**1.09–4.78**

Statistically significant values (*p* < 0.05) are bolded

^a^Grade 0 (no overlapping), Grade 1 (≤ ½ overlapping) and Grade 2 (> ½ overlapping)

^b^Division is based on developmental stages as defined by Nolla’s method (1960)

^c^Dental age in children as assessed by Demirjian’s method (Demirjian et al. 1973; Demirjian and Goldstein 1976) was the same for both maxillary canines

^d^*Frequency too low

Crude logistic regression analysis also showed that a large inclination angle (≥ 25°) was associated with treated canines (OR = 2.41, 95% CI 1.31–4.44), especially in girls (OR = 3.73, 95% CI 1.67–8.34) (Table 2). Only in the girls was a large inclination (≥ 25°) associated with early treatment (Online Appendix 1). A large inclination angle (≥ 25°) was similarly associated with treatment in the late treatment group, especially in the girls (Online Appendix 2).

Adjusted logistic regression showed that treated canines had an association with clear overlapping (OR = 7.18, 95% CI 3.26–15.82), some overlapping (OR = 1.69, 95% CI 1.12–2.55) and a large inclination (≥ 25°) (OR = 2.27, 95% CI 1.12–4.58). Late treatment was likewise associated with both grades of overlapping (Grade 2 OR = 10.43, 95% CI 3.81–28.58 and Grade 1 OR = 2.43, 95% CI 1.37–4.31) and with a large inclination angle (OR = 3.40, 95% CI 1.44–8.02), whereas early treatment was associated only with clear overlapping (OR = 5.41, 95% CI 1.96–14.97) (Table 3).

**Table 3:** Adjusted logistic regression analysis of associations between all the independent variables and treatment need groups in the treated maxillary canines

	Early treatment		Late treatment^d^		All treatments	
	OR	95% CI	OR	95% CI	OR	95% CI
Gender (ref. Girl)						
Boy	0.61	0.34–1.08	2.38	0.95–5.98	0.87	0.56–1.37
Overlapping of canine^a^ (ref. Grade 0)						
Grade 1	1.24	0.72–2.12	**2.43**	**1.37–4.31**	**1.69**	**1.12–2.55**
Grade 2	**5.41**	**1.96–14.97**	**10.43**	**3.81–28.58**	**7.18**	**3.26–15.82**
Inclination of canine (°) (ref. < 15)						
15–19.9	0.53	0.24–1.17	0.92	0.45–1.88	0.69	0.39–1.20
20–24.9	0.94	0.40–2.20	1.53	0.66–3.53	1.15	0.61–2.19
≥ 25	1.61	0.62–4.14	**3.40**	**1.44–8.02**	**2.27**	**1.12–4.58**
Canine root development^b^ (ref. Stage 4)						
Stage 1	0.87	0.15–5.11	**0.15**	**0.03–0.87**	0.38	0.10–1.42
Stage 2	1.52	0.40–5.79	0.41	0.12–1.33	0.85	0.33–2.17
Stage 3	1.17	0.32–4.25	0.39	0.12–1.27	0.70	0.28–1.73
Lateral incisor development^b^ (ref. complete)						
Incomplete	1.28	0.51–3.21	2.53	0.87–7.36	1.60	0.78–3.32
Dental age^c^ (ref. normal)						
Delayed	2.54	0.98–6.60	1.13	0.37–3.50	2.03	0.92–4.49

Statistically significant values (*p* < 0.05) are bolded

^a^Grade 0 (no overlapping), Grade 1 (≤ ½ overlapping) and Grade 2 (> ½ overlapping)

^b^Division is based on developmental stages as defined by Nolla’s method (1960)

^c^Dental age in children as assessed by Demirjian’s method (Demirjian et al. 1973; Demirjian and Goldstein 1976) was the same for both maxillary canines

^d^Adjusted by interaction effect Gender * Lateral incisor development

There were no detected statistical differences between the grades of overlapping and inclination (*p* = 0.053), the stage of canine root development (*p* = 0.558), the stage of lateral incisor development (*p* = 0.656) or dental age (*p* = 0.109) in the treated canines, nor between the inclination of a treated canine and the stage of canine root development (*p* = 0.609), the stage of lateral incisor development (*p* = 0.086) or dental age (*p* = 0.067).

### Developmental stages of the maxillary canines and lateral incisors

The stage of root development could be assessed in 184 treated canines, yielding Nolla’s values in the range 6.5–8.2. There was a statistically significant difference (*p* < 0.001) in the stage of canine root development between the genders, root development being more advanced in the girls. Almost all of the treated canines (84.8%, *n* = 156) had reached one-third to a half of their final length (Stages 2 and 3) at the age of 8.5–10.5 years and their stage of root development did not differ from that observed in a naturally erupted canine (*p* = 0.172) (Table 1). An early stage of canine root development (root formation started, Stage 1) showed a significant inverse association with late treatment in adjusted logistic regression (OR = 0.15, 95% CI 0.03–0.87) (Table 3).

The stage of lateral incisor development could be assessed for 181 teeth, and development was incomplete in 88.4% (*n* = 160) of cases and complete in 11.6% (*n* = 21), with no difference between the genders (*p* = 0.294). The children with treated canine(s) had incomplete lateral incisors (*p* = 0.002) significantly more often than did the children with naturally erupted canines (Table 1), and there was a significant difference when the groups based on treatment needs were compared with the stage of lateral incisor development (*p* = 0.013), almost all the lateral incisors being incomplete in groups receiving interceptive treatment (97.4%, n = 37) and treatment for crowding (93.0%, *n* = 66), whereas a fifth of the lateral incisors had already been assessed as complete in groups receiving early headgear or orthodontic treatment (Table 4).

**Table 4:** Overlapping of canines with the lateral incisor root, inclination (°) of maxillary canines, stages in maxillary canine and lateral incisor root development and dental age vs. need for treatment of maxillary canines

	Interceptive treatment	Early headgear	Orthodontic treatment	Treatment for crowding	*P* value^d^
	*n *(%)	*n* (%)	*n* (%)	*n* (%)	
Overlapping of canine^a^					
Grade 0	11 (30.6)	25 (43.1)	6 (46.2)	20 (28.2)	
Grade 1	19 (52.8)	28 (48.3)	6 (46.2)	41 (57.7)	
Grade 2	6 (16.7)	5 (8.6)	1 (7.7)	10 (14.1)	
Total	36 (100.0)	58 (100.0)	13 (100.0)	71 (100.0)	0.555
Inclination of canine (°)					
< 15	20 (55.6)	41 (70.7)	5 (35.7)	41 (57.7)	
15–19.9	5 (13.9)	5 (8.6)	3 (21.4)	13 (18.3)	
20–24.9	4 (11.1)	8 (13.8)	2 (14.3)	11 (15.5)	
≥ 25	7 (19.4)	4 (6.9)	4 (28.6)	6 (8.5)	
Total	36 (100.0)	58 (100.0)	14 (100.0)	71 (100.0)	0.168
Canine root development^b^					
Stage 1	5 (13.2)	5 (8.6)	2 (14.3)	4 (5.4)	
Stage 2	27 (71.1)	35 (60.3)	6 (42.9)	47 (63.5)	
Stage 3	5 (13.2)	14 (24.1)	6 (42.9)	16 (21.6)	
Stage 4	1 (2.6)	4 (6.9)	0 (0.0)	7 (9.5)	
Total	38 (100.0)	58 (100.0)	14 (100.0)	74 (100.0)	0.310
Lateral incisor development^b^					
Incomplete	37 (97.4)	46 (79.3)	11 (78.6)	66 (93.0)	
Complete	1 (2.6)	12 (20.7)	3 (21.4)	5 (7.0)	
Total	38 (100.0)	58 (100.0)	14 (100.0)	71 (100.0)	0.013
Dental age^c^					
Normal	24 (68.6)	46 (95.8)	14 (100.0)	63 (94.0)	
Delayed	11 (31.4)	2 (4.2)	0 (0.0)	4 (6.0)	
Total	35 (100.0)	48 (100.0)	14 (100.0)	67 (100.0)	< 0.001

^a^Grade 0 (no overlapping), Grade 1 (≤ ½ overlapping) and Grade 2 (> ½ overlapping)

^b^Division is based on developmental stages as defined by Nolla’s method (1960)

^c^Dental age in children as assessed by Demirjian’s method (Demirjian et al. 1973; Demirjian and Goldstein 1976) was the same for both maxillary canines

^d^Fisher’s Exact test

Crude logistic regression showed that an incomplete lateral incisor was associated with treated canines (OR = 2.05, 95% CI 1.18–3.56), especially in girls (OR = 2.51, 95% CI 1.18–5.37) (Table 2); whereas in the cases of early treatment, an incomplete lateral incisor was associated with treatment (Online Appendix 1).

### Dental age

**Fig. 3:**
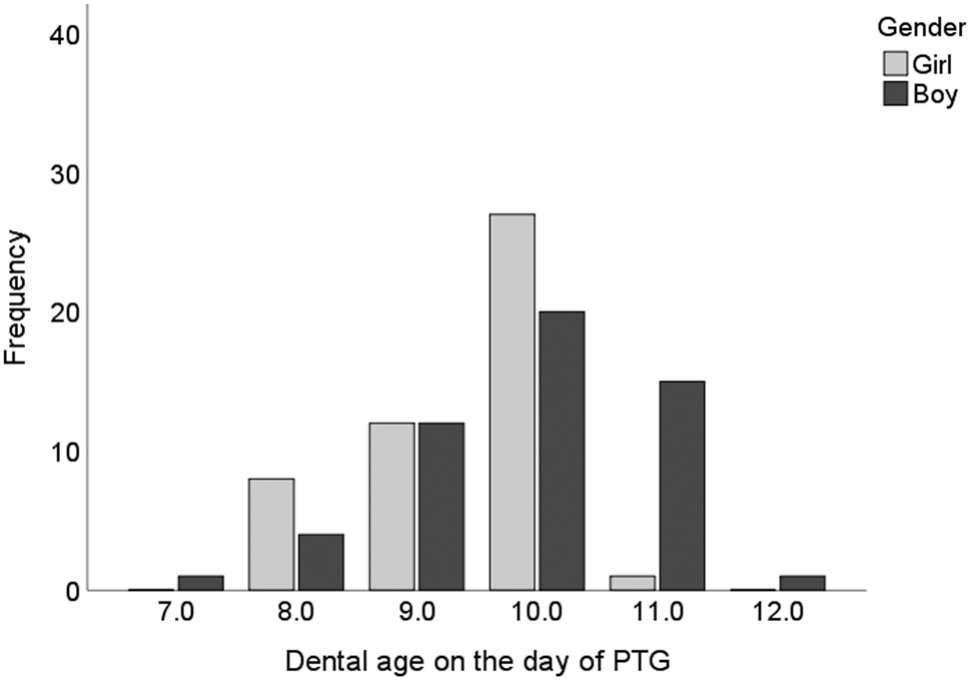
Distribution of children with treated maxillary canine(s) by dental age

Dental age could be assessed from 101 PTGs of the treated children, yielding values in the range 7.1–11.9 years and giving a mean age of 9.6 years (SD 1.0) in all, 9.4 years (SD 0.8) for girls and 9.8 years (SD 1.0) for boys, the gender difference being statistically significant (*p* = 0.021) (Fig. 3). The grouped dental ages were assessed as normal in 90.1% (*n* = 91) of children and delayed in 9.9% (*n* = 10), with no gender difference (*p* = 0.186). The dental age of the treated children was significantly later (*p* = 0.039) than that of the children with naturally erupted canines (9.8 years, SD 0.8) at the time of the PTG, and the children with treated canine(s) had a delayed dental age significantly more often than did the children with naturally erupted canines (*p* = 0.035) (Table 1). There was significant difference when the groups based on treatment needs were compared with those based on dental age (*p* < 0.001) as many as a third (*n* = 11) of the children in the interceptive treatment group has a delayed dental age, whereas almost all the children in the other treatment groups were of normal dental age (Table 4).

Crude logistic regression showed that delayed dental age was associated with treated canines (OR = 2.29, 95% CI 1.09–4.78), albeit no significant association was found in the genders considered separately (Table 2). Delayed dental age was associated with treatment in the cases of early treatment, especially in girls (Online Appendix 1).

## Discussion

Maxillary canine eruption disturbances are common orthodontic problems affecting a developing dentition. We report here on certain features of maxillary permanent canines erupting in the late mixed dentition stage, as seen in PTG, when early or late treatment was subsequently needed to ensure eruption into the oral cavity. The present study and its part I (Ristaniemi et al. 2022) are based on the same representative population and age cohort sample of PTGs from Finnish children aged 8.5–10.5 years. PTG after inspection and palpation is crucial at this age because it gives a view of the maxillary canines a couple of years before their expected eruption into the oral cavity (Eskeli et al. 1999). Furthermore, it provides a retrospective and longitudinal overview of several developmental features affecting the need for treating the maxillary canines, a view which is essentially new and different from that provided by earlier studies (Lindauer et al. 1992; Warford et al. 2003; Sajnani and King 2012a).

The present children who received maxillary canine treatment had overlapping with the lateral incisor more often than did those whose canines erupted naturally, which is in line with the findings of Sajnani and King (2012a). Especially clear overlapping occurred six times more often in the cases with treated canines than in those with naturally erupted canines. A strong association was visible especially in the case of late treatment, where any overlapping was predictive of a treatment need, whereas in early treatment only clear overlapping was associated with a treatment need. Similar associations were also seen in the adjusted logistic regression analyses, thereby verifying the findings.

The children in the present series whose maxillary canines were being treated had significantly larger inclination angles than those whose canines had erupted naturally, and a large inclination (≥ 25°) was observed twice as often in the treated canines. Furthermore, a large inclination (≥ 25°) was significantly related to treatment, particularly in cases of late treatment and in girls. Again, a similar association was seen in the adjusted logistic regression analysis, verifying the findings. Our results are in line with marked inclination of an erupting maxillary canine at the earlier stages of eruption and development (Fernández et al. 1998), after which the canine should upright itself (Sajnani and King 2012a; Ristaniemi et al. 2022).

Warford and co-workers (2003) stated that sectors of overlapping are a better predictive factor than inclination when evaluating the possible impaction of a maxillary canine. Our findings regarding treated canines as seen in PTG at the age 8.5–10.5 years support that observation and underline the importance of overlapping as a predictive feature of later treatment needs. Nevertheless, a large maxillary canine inclination angle at the age of 8.5–10.5 years was also found here to be an important predictive feature for later treatment needs, which confirms earlier findings of larger inclination angles in abnormal maxillary canine eruption paths (Sajnani and King 2012a; Chalakkal et al. 2011).

The present observation that the stage in maxillary canine root development did not differ between the treated and naturally erupted maxillary canines agrees with the results of one earlier study (Sajnani and King 2012a). The stage of canine root development did not come to the fore as being predictive of treatment needs, even though an early stage of development did reduce the need for late treatment. The development stage of the treated canines at the age of 8.5–10.5 years did differ between the genders, however, being more advanced in the girls than in the boys, which agrees with the general understanding and with earlier findings of gender differences (Haavikko 1970). On the other hand, we did not find any gender differences in the stage of lateral incisor development in the children with treated canine(s), in contrast to the situation in the group of naturally erupted maxillary canines (Ristaniemi et al. 2022), where the development of both teeth was considered to be more advanced in the girls.

It was found here that children with treated maxillary canine(s) significantly more often had incomplete lateral incisors than did those with naturally erupted canines at the age of 8.5–10.5 years. Fernández and his research group (1998) stated that the overlapping of a maxillary canine with a lateral incisor root can be an early sign of abnormal eruption of the canine if the development of the lateral incisor is complete. In contrast to that finding, only one out of the ten children in our series with treated canine(s) had complete lateral incisors, whereas complete lateral incisors were found twice as often in the group of children with naturally erupted canines (Ristaniemi et al. 2022). Furthermore, an incomplete lateral incisor was significantly related to the treatment need, especially in the girls and all children in the early treatment group.

In the present children, the exact dental age was considered to be earlier in the boys with treated maxillary canine(s) than in the corresponding girls, which is congruent with the results quoted in part I (Ristaniemi et al. 2022) but differs from those of earlier studies (Demirjian and Levesque 1980; Chaillet et al. 2004). Although the boys were considered to be significantly more advanced in their dental age at the physical age of 8.5–10.5 years, the stage of canine root development was more advanced in the girls. Children with treated canine(s) are reported to have a delayed dental age significantly more often than do children with naturally erupted canines, which is in line with earlier findings that late timing of dental development is related to displacement of a maxillary canine (Peck 2009; Rozylo-Kalinowska et al. 2011; Naser et al. 2011). In our results, delayed dental age was related to treatment, especially to early treatment in girls. One interesting finding was that a third of the children in the interceptive treatment group had delayed dental age, so that this differs clearly from other treatment groups, where almost all of the children had early/normal dental age.

Sajnani and King (2012a) showed that displacement can be diagnosed early, from the age of eight onwards. We have shown previously that a maxillary canine can erupt naturally despite overlapping and a large inclination angle at this age (Ristaniemi et al. 2022). In fact, the present results show that overlapping, especially clear overlapping, and a large inclination angle seen in PTG at the age of 8.5–10.5 years can be an early sign of a treatment need and ought to be monitored. Our findings are in agreement with Ericson and Kurol (1986), who maintain that disturbances in the eruption of maxillary canines cannot be diagnosed radiographically in children younger than 10 years because of the large variation and high chances of spontaneous correction of the eruption path.

In this sample from the normal population aged 8.5–10.5 years, a small number of children eventually needed treatment for the eruption of a maxillary canine and all the treated canines eventually erupted into the oral cavity. Every tenth maxillary canine needed some treatment, including early treatment procedures to create more space in the dental arch for their eruption.

Early headgear commencing between the early and late mixed dentition stages is commonly the choice for early treatment in Finland and has been shown to have a positive effect on the eruption paths of the maxillary canines (Silvola et al. 2009; Hadler-Olsen et al. 2018). In the present study, treatment with early headgear was enough for a third of these canines, which indicates its ability to improve the eruption path. Another notable finding in the early treatment group was that the children receiving interceptive treatment considerably more often had an incomplete lateral incisor and a delayed dental age. A deciduous canine is usually extracted after negative palpation in the labial sulcus and malposition of the canine observed in PTG.

The most common option for the present children was treatment for crowding, which underlines the importance of creating space for the developing dentition. Furthermore, incomplete lateral incisors were significantly emphasised in the treatment for the crowding group. Monitoring the eruption of maxillary lateral incisors and canines at the individual level during the latent period after the first mixed stage of the dentition is a possibility for early treatment and can be expected to have an effect on space conditions in the dental arch.

One limitation of this work can be seen in the study design, which is retrospective, in addition to which the material was collected from clinical dental records, which were not all complete in either the health centre’s paper archives or in the software after the PTG. The need for treatment of maxillary canines in subsamples was substantial small, a situation which may have affected the results.

Our results showed that the maxillary canines that were treated were significantly more often overlapping with a lateral incisor and had larger inclination angles than the naturally erupted maxillary canines. Thus, this hypothesis is confirmed. The second hypothesis was that root development in the maxillary canines and lateral incisors is in an earlier stage and dental age is late relative to children with naturally erupted maxillary canines. Our results showed that the root development stage of the maxillary canines studied here did not differ from that of naturally erupted maxillary canines, so that this part of the hypothesis must be rejected. However, treated canines more often had incomplete lateral incisors and delayed dental age, hence this part of hypothesis can be accepted. Overall, eruption of a maxillary canine is always a unique event, and the importance of monitoring by palpation and radiography as well as ensuring space in the dental arch during the mixed stage of the dentition should never be underestimated.

The clinical aspect adopted here by estimating the eruption of maxillary canines for a couple of years before their actual eruption into the oral cavity and assessing the prospective need for treatment by searching for associations between developmental features of maxillary canine and the actual treatment needs occurring later is new. Similar studies will be needed in future to fill in the scientific background and gather data to support clinical decision-making. These results should help a dentist to decide when to monitor the situation in order to ensure the provision of adequate space in the dental arch.

## Conclusions

Considering the limitations of the present study the following conclusions can be made:

- In this cohort aged 8.5–10.5 years derived from a normal population, about ten percent of canines eventually needed treatment to erupt into the oral cavity, including interceptive treatment procedures.
- Canines that were treated later had overlapping, a larger inclination and incomplete lateral incisors significantly more often than did naturally erupted canines, and the children concerned significantly more often had a delayed dental age.
- Clear overlapping of a maxillary canine with a lateral incisor was closely associated with treatment, regardless of gender, whereas some degree of overlapping seemed to be associated with late treatment.
- A marked inclination angle was associated with treatment, especially in girls, and this was emphasised in the late treatment children.
- An incomplete lateral incisor was associated with treatment, especially in girls.
- A delayed dental age was associated with treatment, especially in the early treatment girls.
- Monitoring the space conditions at the individual level already after the first mixed stage will enable early treatment.

## Supplementary Information

Below is the link to the electronic supplementary material.

Supplementary file1 (DOCX 21 KB)

## Data Availability

Not applicable.
