# Supplementary material for: Eruption pattern of the maxillary canines: features indicating treatment needs as seen in PTG at the late mixed stage—Part II
Source: Eur Arch Paediatr Dent. 2022 Jun 10;23(4):567–78. doi: 10.1007/s40368-022-00719-5 (PMC9337999; doi:10.1007/s40368-022-00719-5)
Supplement: Supplementary file 1 — Supplementary file1 (DOCX 21 KB) [file 40368_2022_719_MOESM1_ESM.docx]

**Eruption pattern of the maxillary canines: features indicating treatment needs as seen in PTG at the late mixed stage****–Part II**

**European Archives of Paediatric Dentistry**

Jenni Ristaniemi^1^, Terhi Karjalainen^1^, Kati Kujasalo^1^, Wille Rajala^1^, Paula Pesonen^2^, Raija Lähdesmäki^1,3^

^1^ Research Unit of Oral Health Sciences, Oral Development and Orthodontics, Faculty of Medicine, University of Oulu, Oulu, Finland

^2^ Infrastructure for Population Studies, Faculty of Medicine, University of Oulu, Oulu, Finland

^3^ Oral and Maxillofacial Department, Medical Research Center Oulu (MRC Oulu), Oulu University Hospital, Oulu, Finland

**Correspondence**

Jenni Ristaniemi

E-mail: jenni.ristaniemi@oulu.fi

**Appendix 1** Crude logistic regression analysis of associations between the independent variables and early treatment by gender in the treated maxillary canines. Statistically significant values (p < 0.05) are bolded

|  | **Girls** | | **Boys** | | **All** | | |
| --- | --- | --- | --- | --- | --- | --- | --- |
|  | **OR** | **95% CI** | **OR** | **95% CI** | **OR** | **95% CI** |  |
| **Overlapping of canine^a^ (ref. Grade 0)** |  |  |  |  |  |  |  |
| Grade 1 | 1.52 | (0.80-2.89) | 1.28 | (0.62-2.64) | 1.44 | (0.89-2.32) |  |
| Grade 2 | **3.73** | **(1.07-12.9)** | **10.97** | **(2.67-45.03)** | **6.13** | **(2.45-15.33)** |  |
| **Inclination of canine (⁰) (ref. < 15)** |  |  |  |  |  |  |  |
| 15 - 19.9 | 0.65 | (0.25-1.70) | 0.61 | (0.22-1.72) | 0.63 | (0.31-1.28) |  |
| 20 - 24.9 | 1.53 | (0.60-3.86) | 0.57 | (0.14-2.27) | 1.07 | (0.51-2.27) |  |
| ≥ 25 | **3.12** | **(1.19-8.18)** | 0.75 | (0.14-3.90) | 1.99 | (0.90-4.39) |  |
| **Canine root development^b^ (ref. Stage 4)** |  |  |  |  |  |  |  |
| Stage 1 | *^d^ | * | 4.04 | (0.42-38.61) | 2.71 | (0.74-9.99) |  |
| Stage 2 | * | * | 1.95 | (0.23-16.68) | 2.20 | (0.75-6.48) |  |
| Stage 3 | * | * | 1.21 | (0.12-12.00) | 1.59 | (0.51-4.97) |  |
| **Lateral incisor development^b^ (ref. complete)** |  |  |  |  |  |  |  |
| Incomplete | 2.09 | (0.86-5.08) | 2.75 | (0.68-11.09) | **2.10** | **(1.01-4.39)** |  |
| **Dental age^c^ (ref. normal)** |  |  |  |  |  |  |  |
| Delayed | **3.48** | **(1.17-10.34)** | 1.81 | (0.36-9.04) | **2.94** | **(1.22-7.11)** |  |

^a^ Grade 0 (no overlapping), Grade 1 (≤ ½ overlapping) and Grade 2 (> ½ overlapping)

^b^ Division is based on developmental stages as defined by Nolla’s method (1960)

^c^ Dental age in children as assessed by Demirjian’s method (Demirjian et al. 1973, Demirjian and Goldstein 1976) was the same for both maxillary canines

^d^ *, frequency too low

**Appendix 2** Crude logistic regression analysis of associations between the independent variables and late treatment by gender in the treated maxillary canines. Statistically significant values (p < 0.05) are bolded

|  | **Girls** | | **Boys** | | **All** | | |
| --- | --- | --- | --- | --- | --- | --- | --- |
|  | **OR** | **95% CI** | **OR** | **95% CI** | **OR** | **95% CI** |  |
| **Overlapping of canine^a^ (ref. Grade 0)** |  |  |  |  |  |  |  |
| Grade 1 | **2.96** | **(1.32-6.63)** | **2.49** | **(1.27-4.89)** | **2.65** | **(1.58-4.45)** |  |
| Grade 2 | **9.25** | **(2.57-33.29)** | **13.01** | **(3.60-47.08)** | **10.30** | **(4.23-25.12)** |  |
| **Inclination of canine (⁰) (ref. < 15)** |  |  |  |  |  |  |  |
| 15 - 19.9 | 1.32 | (0.53-3.28) | 1.34 | (0.62-2.89) | 1.32 | (0.73-2.38) |  |
| 20 - 24.9 | 2.01 | (0.69-5.80) | 1.48 | (0.56-3.93) | 1.69 | (0.83-3.46) |  |
| ≥ 25 | **4.14** | **(1.42-12.03)** | 1.96 | (0.63-6.14) | **2.86** | **(1.33-6.16)** |  |
| **Stage of canine root development^b^ (ref. Stage 4)** |  |  |  |  |  |  |  |
| Stage 1 | *^d^ | * | 0.85 | (0.17-4.15) | 0.80 | (0.23-2.73) |  |
| Stage 2 | * | * | 1.09 | (0.29-4.04) | 1.13 | (0.49-2.59) |  |
| Stage 3 | * | * | 0.56 | (0.13-2.48) | 0.83 | (0.34-2.07) |  |
| **Stage of lateral incisor development^b^ (ref. complete)** |  |  |  |  |  |  |  |
| Incomplete | 3.24 | (1.00-10.55) | 1.21 | (0.49-3.03) | 1.92 | (0.94-3.91) |  |
| **Dental age^c^ (ref. normal)** |  |  |  |  |  |  |  |
| Delayed | * | * | 1.77 | (0.46-6.88) | 1.44 | (0.52-3.97) |  |

^a^ Grade 0 (no overlapping), Grade 1 (≤ ½ overlapping) and Grade 2 (> ½ overlapping)

^b^ Division is based on developmental stages as defined by Nolla’s method (1960)

^c^ Dental age in children as assessed by Demirjian’s method (Demirjian et al. 1973, Demirjian and Goldstein 1976) was the same for both maxillary canines

^d^ *, frequency too low
